# Mutations in troponin T associated with Hypertrophic Cardiomyopathy increase Ca^2+^-sensitivity and suppress the modulation of Ca^2+^-sensitivity by troponin I phosphorylation^☆^

**DOI:** 10.1016/j.abb.2016.03.027

**Published:** 2016-07-01

**Authors:** Andrew E. Messer, Christopher R. Bayliss, Mohammed El-Mezgueldi, Charles S. Redwood, Douglas G. Ward, Man-Ching Leung, Maria Papadaki, Cristobal dos Remedios, Steven B. Marston

**Affiliations:** a Imperial College London, London, UK; b University of Leicester, Leicester, UK; c University of Oxford, Oxford, UK; d University of Birmingham, Birmingham, UK; e University of Sydney, Sydney, Australia

**Keywords:** Hypertrophic Cardiomyopathy, Troponin T, Phosphorylation of troponin I, Ca^2+^ regulation of contractility, *In vitro* motility assay, DCM, Dilated Cardiomyopathy, EGCG, Epigallocatechin-3-Gallate, HCM, Hypertrophic Cardiomyopathy, IVMA, *In vitro* Motility Assay, k_ACT_, Rate of force development, k_REL_, Rate of fast relaxation phase, n_H_, Hill Coefficient, TnC, Troponin C, TnI, Troponin I, TnT, Troponin T, WT, wild-type

## Abstract

We investigated the effect of 7 Hypertrophic Cardiomyopathy (HCM)-causing mutations in troponin T (TnT) on troponin function in thin filaments reconstituted with actin and human cardiac tropomyosin. We used the quantitative *in vitro* motility assay to study Ca^2+^-regulation of unloaded movement and its modulation by troponin I phosphorylation. Troponin from a patient with the K280N TnT mutation showed no difference in Ca^2+^-sensitivity when compared with donor heart troponin and the Ca^2+^-sensitivity was also independent of the troponin I phosphorylation level (uncoupled). The recombinant K280N TnT mutation increased Ca^2+^-sensitivity 1.7-fold and was also uncoupled. The R92Q TnT mutation in troponin from transgenic mouse increased Ca^2+^-sensitivity and was also completely uncoupled. Five TnT mutations (Δ14, Δ28 + 7, ΔE160, S179F and K273E) studied in recombinant troponin increased Ca^2+^-sensitivity and were all fully uncoupled. Thus, for HCM-causing mutations in TnT, Ca^2+^-sensitisation together with uncoupling *in vitro* is the usual response and both factors may contribute to the HCM phenotype. We also found that Epigallocatechin-3-gallate (EGCG) can restore coupling to all uncoupled HCM-causing TnT mutations. In fact the combination of Ca^2+^-desensitisation and re-coupling due to EGCG completely reverses both the abnormalities found in troponin with a TnT HCM mutation suggesting it may have therapeutic potential.

## Highlights

• 7 HCM-causing mutations in cardiac TnT were studied using *in vitro* motility assay.
• All the mutations increased myofilament Ca^2+^-sensitivity (range 1.5–2.7 fold).
• All mutations suppressed the modulation of Ca^2+^-sensitivity by TnI phosphorylation.
• Epigallocatechin-3-gallate (EGCG) restored this modulation to all mutations.
• This suggests a therapeutic potential for EGCG in the treatment of HCM.

## 1 Introduction

Hypertrophic cardiomyopathy (HCM) is the most common inherited cardiomyopathy and is usually associated with mutations in sarcomeric proteins. A recent study has shown that 11% of the identified mutations are in the proteins of the thin filament; actin, tropomyosin, troponin I (TnI), troponin C (TnC) and troponin T (TnT) [1]. It is generally found that HCM-causing mutations result in a 2–3 fold higher myofilament Ca^2+^-sensitivity compared to normal heart muscle and this has been proposed to be necessary to trigger the symptoms of HCM: a hyper-contractile phenotype, heart muscle hypertrophy, myocyte disarray and fibrosis [2], although the clinical manifestation of HCM is very variable, probably due to background genetic and environmental factors.

Mutations in the same sarcomeric protein genes are also associated with familial dilated cardiomyopathy (DCM), a disease characterised by a hypo-contractile phenotype with dilation of the heart chamber and thinning of cardiac muscle [3]. Whilst DCM clearly has a separate molecular mechanism from HCM, the simple hypothesis that Ca^2+^-sensitivity is reduced in familial DCM (the opposite of HCM) has proven to be incorrect since changes of Ca^2+^-sensitivity do not correlate with the phenotype [2], [4], [5]. Our investigations have established that a consistent feature of DCM-causing mutations in thin filament proteins is that the Ca^2+^-sensitivity is not modulated by TnI phosphorylation, a process we have called uncoupling, and we have proposed that this is a disease-mechanism [4], [5], [6].

TnI is one of several substrates of protein kinase A (PKA). In normal heart, on adrenergic stimulation PKA is activated and TnI phosphorylation at Ser22 and 23 is increased. Phosphorylation of TnI decreases myofibrillar Ca^2+^-sensitivity 2–3 fold and correspondingly increases the rate of Ca^2+^ dissociation from TnC. This is an essential component of the lusitropic response to adrenergic stimulation, consequently DCM mutations cause a blunting of the response to β1 agonists, and this leads to a reduced cardiac reserve and predisposes the heart to failure under stress [6].

Mutations in the cardiac TnT gene (*TNNT2*) are the most common HCM-causing mutations after *MYH7* and *MYBPC3*. There have been persistent reports from *in vitro* studies that HCM-causing mutations in the other thin filament proteins: TnI, TnC, actin and tropomyosin uncouple changes in myofilament Ca^2+^-sensitivity from TnI phosphorylation level in addition to increasing absolute Ca^2+^-sensitivity (reviewed by Messer [5] and refs therein [7], [8], [9], [10], [11], [12], [13], [14], [15], [16], [17]). However there have not been any systematic studies of mutations in TnT associated with HCM. It is possible that uncoupling is a consistent consequence of HCM-causing mutations in all thin filament proteins and if so, it could contribute to the HCM phenotype.

In this study we investigate the relationship of myofilament Ca^2+^-sensitivity to TnI phosphorylation in 7 HCM related mutations in human cardiac TnT by quantitative *in vitro* motility assay. Six of these are well-established mutations, whereas the seventh is a unique human heart sample from an HCM patient with a homozygous mutation, K280N. All these mutations induced uncoupling, thus extending the range of cardiac disease associated with uncoupling and raising the possibility of a physiological role in accounting for the HCM phenotype.

## 2 Methods

### 2.1 Sources of contractile proteins

Troponin was isolated from human or mouse heart muscle using an anti-cardiac troponin I (TnI) monoclonal antibody affinity column as described [18]. We used heart muscle from TnT R92Q transgenic mice and non-transgenic littermates [19] and explanted heart from a patient with a homozygous TnT K280N mutation with donor heart muscle as control [20]. Donor hearts had no history of cardiac disease and normal ECG and ventricular function and were obtained when no suitable transplant recipient was found. Approval was granted by the Human Research Ethics Committees of both the University of Sydney (Protocol No. 2814) and St. Vincent's Hospital (Protocol No. H91/048/1a) for collection and distribution of the human heart samples and by the NHS National Research Ethics Service, South West London REC3 (10/H0803/147) for study of the samples. Patients gave written consent with PIS approved by the relevant ethical committee. All samples are anonymised. The investigations conform to the principles of the Declaration of Helsinki. Recombinant human cardiac TnT K280N (T3 isoform) was introduced into donor heart troponin by exchange as described [18]. Recombinant whole troponin incorporating HCM-causing mutations in TnT (T3 isoform) was prepared as described [21], [22].

### 2.2 Manipulation and measurement of TnI phosphorylation level

Troponin isolated from human heart samples has a high level of phosphorylation, which was reduced by treatment with shrimp alkaline phosphatase (Sigma P9088). Recombinant TnI was phosphorylated by treatment with protein kinase A (PKA) catalytic subunit (Sigma, P2645-400) as previously described [4], [23]. TnI phosphorylation levels in isolated troponin were measured by phosphate affinity SDS-PAGE as described by Messer et al. [24].

### 2.3 Quantitative *in vitro* motility assay (IVMA)

Thin filaments were reconstituted with 10 nM rabbit skeletal or mouse cardiac muscle α-actin (labelled with TRITC phalloidin), tropomyosin (40–60 nM) and troponin (60 nM) to study Ca^2+^-regulation of filament motility by the quantitative *in vitro* motility assay (IVMA) [4], [18], [25]. Thin filament movement over a bed of immobilised rabbit fast skeletal muscle heavy meromyosin (100 μg/ml) was compared in two channel motility cells in which troponin varied by a single factor (mutation, phosphorylation state or treatment with drug). The temperature was set to 29 °C. Filament movement was recorded and analysed as previously described [26], yielding two parameters, the fraction of filaments moving and the speed of moving filaments.

The fraction motile and the sliding speeds were measured over a range of Ca^2+^ concentrations to generate Ca^2+^-activation curves as shown previously [4], [17]. The data were fitted to the 4-variable Hill equation to yield a value for EC_50_ and n_H_. EC_50_ values from replicate experiments were analysed by paired *t*-test since the distribution of EC_50_ has been shown to be normal.

## 3 Results

### 3.1 Human heart sample with TnT K280N mutation

We initially studied a TnT mutation in a unique cardiac muscle sample from a patient with HCM due to a homozygous mutation in TnT, K280N. The patient had a septal myectomy but subsequently required a heart transplant at age 26. The mutation was identified and contractility of skinned myocytes was investigated in a previous study [27].

We isolated troponin from the sample and confirmed that asparagine had exclusively replaced a lysine at amino acid 280 (K280N) by mass spectrometry (Supplemental data). The TnI phosphorylation, measured by phosphate affinity SDS-PAGE, was in the range 1.4–1.6 mols Pi/mol TnI. This is not significantly different from donor heart troponin (Table 1) and substantially greater than heart samples from septal myectomy operations, indicating that the myectomy operation had restored phosphorylation levels to normal although this did not prevent subsequent heart failure requiring heart transplant [27].

**Table 1:** Effect of the HCM related TnT K280N mutation on Ca^2+^-sensitivity and phosphorylation dependence of Ca^2+^-sensitivity using the TnT K280N patient sample (see Fig. 1). X = exchanged. All data rounded to 2 significant figures.

	Experiment	EC_50_, μM		Ratio (EC_50_ Test/Control)	n	p	TnI Phosphorylation	
	Test vs Control	Test	Control				Test	Control
A	Donor vs K280N patient	0.19 ± 0.015	0.20 ± 0.016	×0.99 ± 0.047	11	0.87	1.6 ± 0.060	1.4 ± 0.020
B	K280N vs dpK280N patient	0.14 ± 0.022	0.14 ± 0.024	×0.99 ± 0.020	6	0.54	1.4 ± 0.090	0.11 ± 0.10
C	Donor vs dpDonor	0.14 ± 0.030	0.050 ± 0.010	×3.1 ± 0.55	10	0.0040	1.6 ± 0.070	0.23 ± 0.070
D	K280N patientXTnT vs dpK280N patientXTnT	0.16 ± 0.035	0.088 ± 0.013	×1.8 ± 0.17	4	0.022	1.4 ± 0.12	0.090 ± 0.070

**Fig. 1:**
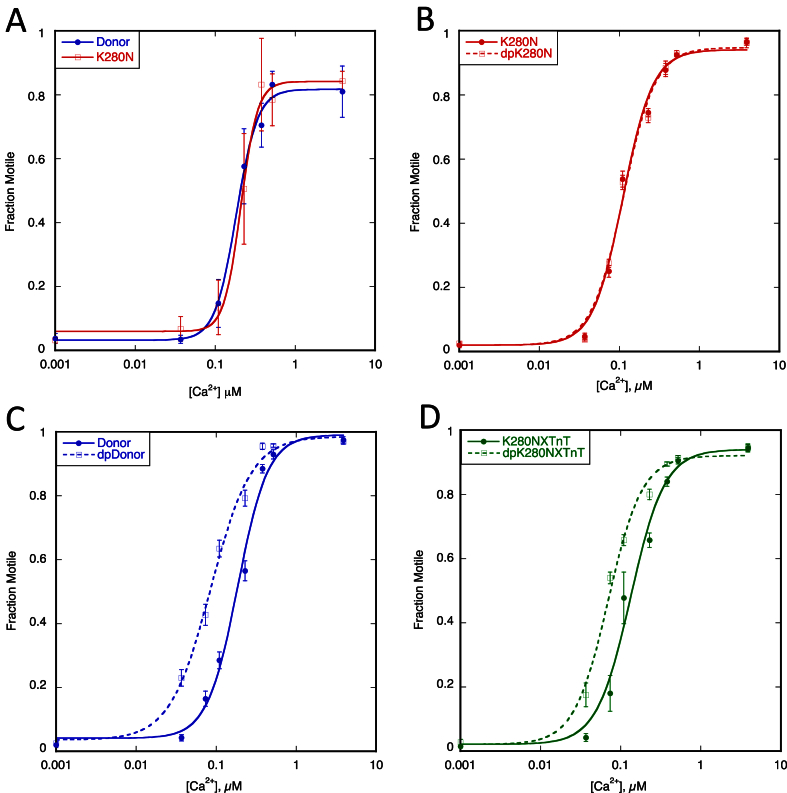
**Effect of the HCM related TnT K280N mutation (patient sample)**. The fraction of filaments motile, measured in a paired experiment by IVMA, is plotted against [Ca^2+^] for representative single experiments. The mean values of EC_50_ from replicate experiments are shown in Table 1. Solid lines and points, phosphorylated troponin; dotted lines and open points, unphosphorylated troponin. Error bars represent SEM of four measurements of motility in the same motility chamber. Blue, native donor thin filaments; red, TnT K280N patient thin filaments; green, WT TnT exchanged into TnT K280N patient thin filaments. A. Donor and TnT K280N patient containing thin filaments: no change in EC_50_ (Ca^2+^-sensitivity) was seen. B. Effect of phosphorylation on thin filaments containing the TnT K280N mutation. The relationship of Ca^2+^-sensitivity to TnI phosphorylation is uncoupled. C. Effect of phosphorylation on donor thin filaments. Normal relationship as phosphorylation increased EC_50_. D. The difference in Ca^2+^-sensitivity is restored when recombinant human cardiac TnT was exchanged into native TnT K280N patient thin filaments.

We investigated the effect of the TnT K280N mutation on troponin function in thin filaments reconstituted with actin and human cardiac tropomyosin. We used IVMA to study thin filament Ca^2+^-regulation of unloaded movement over immobilised heavy meromyosin. When we compared thin filaments containing donor heart and TnT K280N patient heart troponin there was no difference in the Ca^2+^-sensitivity or maximum sliding speed (Fig. 1, Table 1).

**Fig. 2:**
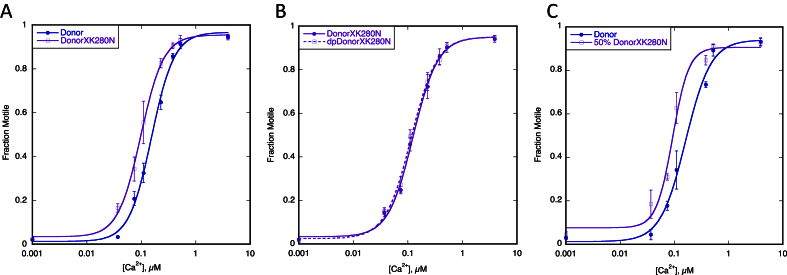
**Effect of the HCM related TnT K280N mutation (recombinant protein)**. The fraction of filaments motile, measured in a paired experiment by IVMA, is plotted against [Ca^2+^] for representative single experiments. The mean values of EC_50_ from replicate experiments are shown in Table 2. Solid lines and points, phosphorylated troponin; dotted lines and open points, unphosphorylated troponin. Error bars represent SEM of four measurements of motility in the same motility chamber. Blue, native donor thin filaments; purple, donor thin filaments exchanged with recombinant human cardiac TnT K280N. A. Thin filaments containing donor troponin and donor troponin exchanged with recombinant human cardiac TnT K280N show the difference in EC_50_ (Ca^2+^-sensitivity) normally seen with HCM-causing mutations. B. Effect of phosphorylation on exchanged thin filaments containing the recombinant human cardiac TnT K280N. The relationship of Ca^2+^-sensitivity to TnI phosphorylation is uncoupled. C. Effect of 50% mutation. The difference in EC_50_ (decreased Ca^2+^ sensitivity) is also seen with 50% exchanged thin filaments.

We previously found that this pattern of results was typical of muscle from the interventricular septum of hypertrophic obstructive cardiomyopathy (HOCM) patients that generally also had an uncoupled relationship between Ca^2+^-sensitivity and TnI phosphorylation accompanied by very low levels of TnI phosphorylation [23]. We therefore determined the effect of dephosphorylating the TnT K280N patient troponin on Ca^2+^-regulation. In contrast to the 3.1-fold increase in Ca^2+^-sensitivity of donor heart troponin when it was dephosphorylated, the Ca^2+^-sensitivity of thin filaments containing TnT K280N patient troponin was independent of the level of phosphorylation (Fig. 2, Table 1). We then replaced the TnT in the TnT K280N patient sample with recombinant wild-type TnT by an exchange reaction (exchange was 89% [23]) and observed that the dependence of Ca^2+^-sensitivity on TnI phosphorylation was restored.

In a previous study of troponin from patients with obstructive HCM, we found that the myofilament Ca^2+^-sensitivity was similar to donor heart troponin, as observed here, and did not change with TnI phosphorylation level independently of the gene that was mutated [23]. In order to determine the direct effects of the TnT K280N mutation, we investigated the effect of recombinant human cardiac TnT K280N on thin filament regulation. Comparison of donor heart troponin and donor heart troponin with the TnT component replaced by TnT K280N showed that the mutation increased Ca^2+^-sensitivity 1.7-fold; a similar shift in Ca^2+^-sensitivity was obtained with 90% and 45% exchanged (Fig. 2, Table 2). However, the Ca^2+^-sensitivity of troponin containing recombinant TnT K280N did not depend on TnI phosphorylation. The increased Ca^2+^-sensitivity due to recombinant TnT K280N is typical of HCM-causing mutations *in vitro* and indicates that the absence of Ca^2+^-sensitivity shift in the patient sample was probably due to secondary abnormalities similar to troponin from the IVS of HOCM patients.

**Table 2:** Effect of the HCM related TnT K280N mutation on Ca^2+^-sensitivity and phosphorylation dependence of Ca^2+^-sensitivity using recombinant TnT K280N (see Fig. 2). X = exchanged. All data rounded to 2 significant figures.

	Experiment	EC_50_, μM		Ratio (EC_50_ Test/Control)	N	p	TnI Phosphorylation	
	Test vs Control	Test	Control				Test	Control
A	Donor vs DonorXK280N	0.17 ± 0.037	0.10 ± 0.020	×1.7 ± 0.12	6	0.0028	1.6 ± 0.11	1.6 ± 0.11
B	DonorXK280N vs dpDonorXK280N	0.10 ± 0.0050	0.098 ± 0.0050	×1.1 ± 0.037	10	0.11	1.6 ± 0.060	0.34 ± 0.19
C	Donor v 50% DonorXK280N	0.14 ± 0.018	0.089 ± 0.0080	×1.6 ± 0.13	4	0.018	1.6 ± 0.11	1.6 ± 0.11

### 3.2 HCM-causing TnT mutations studied in transgenic mice

**Fig. 3:**
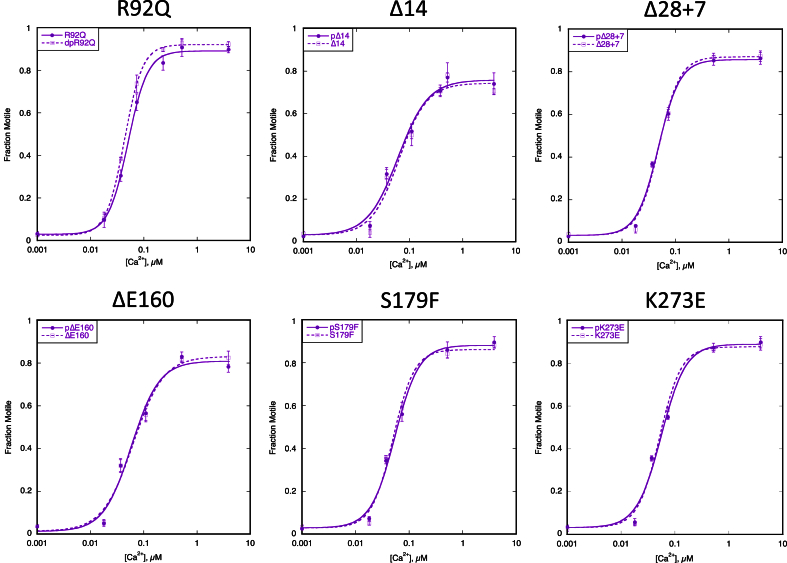
**Effect of the troponin I phosphorylation on Ca**^**2+**^**-sensitivity of thin filaments containing HCM-causing TnT mutations**. The fraction of filaments motile, measured in a paired experiment by IVMA, is plotted against [Ca^2+^] for representative single experiments. The mean values of EC_50_ from replicate experiments are shown in Table 3. Solid lines and points, phosphorylated troponin; dotted lines and open points, unphosphorylated troponin. Error bars represent SEM of four measurements of motility in the same motility chamber. Purple, HCM-causing TnT mutation thin filaments. The mean values of EC_50_ from replicate experiments is plotted in Fig. 4B and summarised in Table 3. All of the HCM-causing mutations tested show uncoupling in the IVMA when phosphorylated and unphosphorylated thin filaments are compared.

**Fig. 4:**
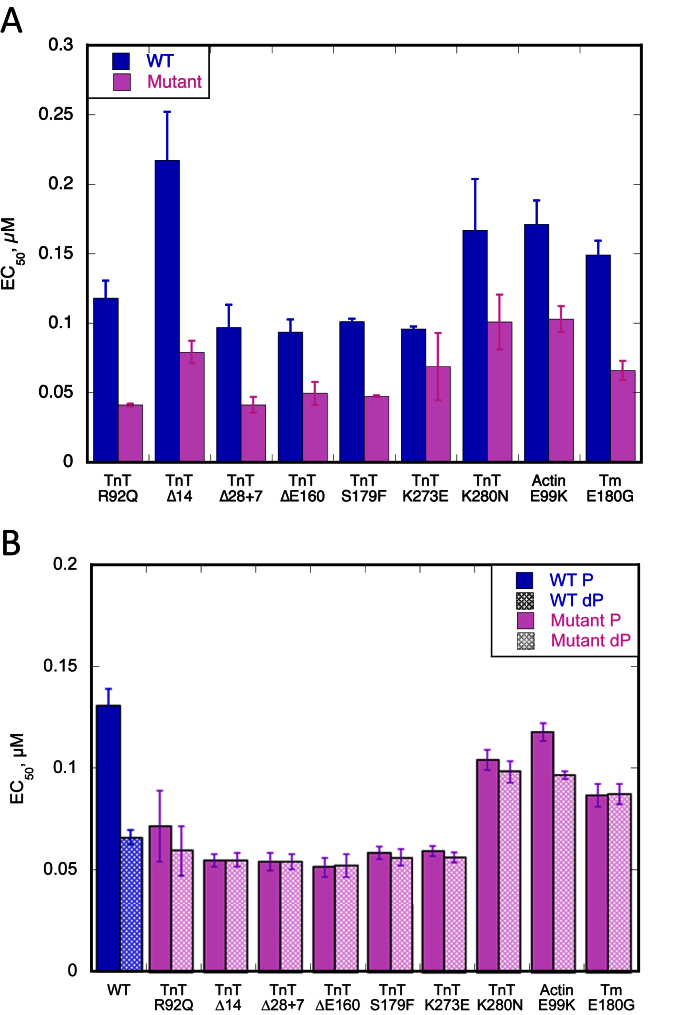
**Effect of the different HCM-causing TnT mutations on Ca**^**2+**^**-sensitivity and its modulation by TnI phosphorylation**. Summary of the HCM-causing TnT mutations. Mean EC_50_ values from replicate paired experiments are plotted, see Table 3. Error bars are ± SEM. Blue, WT thin filaments; Blue hatched, unphosphorylated WT thin filaments; Purple, HCM-causing TnT mutant thin filaments; Purple hatched, unphosphorylated mutant thin filaments. The previously studied *ACTC* E99K and *TPM1* E180G were included as a comparison. A. All the HCM-causing TnT mutants show increased Ca^2+^-sensitivity (decreased EC_50_) compared to WT thin filaments. B. All the HCM-causing TnT mutants are uncoupled in comparison to WT thin filaments.

We next examined the HCM-related TnT mutation R92Q in the tropomyosin binding domain of TnT expressed in mouse models [19]. The level of mutation expression in these lines is 67%. Compared to troponin from non-transgenic mouse hearts, the TnT R92Q mutation increased Ca^2+^-sensitivity and uncoupled the relationship between Ca^2+^-sensitivity and TnI phosphorylation (Fig. 3, Fig. 4, Table 3) [17].

**Table 3:** Effect of the HCM related TnT mutations on Ca^2+^-sensitivity and phosphorylation dependence of Ca^2+^-sensitivity (see Fig. 3, Fig. 4). All data rounded to 2 significant figures.

TnT mutation	EC_50_ WT μM ± SEM	EC_50_ mutant μM ± SEM	Ratio EC_50_ WT/mutant ± SEM	EC_50_ mutant P μM ± SEM	EC_50_ mutant dP μM ± SEM	Ratio EC_50_ P/dP
R92Q	0.12 ± 0.013	0.041 ± 0.00091	2.9 ± 0.28	0.072 ± 0.017	0.060 ± 0.012	1.1 ± 0.045
Δ14	0.22 ± 0.035	0.079 ± 0.0081	2.7 ± 0.16	0.055 ± 0.0031	0.055 ± 0.0032	1.0 ± 0.019
Δ28 + 7	0.097 ± 0.016	0.041 ± 0.0057	2.3 ± 0.068	0.054 ± 0.0041	0.055 ± 0.0037	1.0 ± 0.015
ΔE160	0.094 ± 0.0091	0.049 ± 0.0082	2.0 ± 0.30	0.052 ± 0.0046	0.053 ± 0.0056	1.0 ± 0.043
S179F	0.10 ± 0.0020	0.047 ± 0.00049	2.1 ± 0.020	0.059 ± 0.0030	0.057 ± 0.0038	1.1 ± 0.096
K273E	0.096 ± 0.0019	0.069 ± 0.024	1.6 ± 0.53	0.059 ± 0.0024	0.056 ± 0.0026	1.1 ± 0.014
K280N	0.17 ± 0.037	0.10 ± 0.020	1.7 ± 0.12	0.10 ± 0.0048	0.098 ± 0.0052	1.1 ± 0.037

### 3.3 HCM-causing TnT mutations studied using recombinant troponin

A further five HCM-causing mutations in TnT were examined using recombinant troponin expressed as the whole troponin complex in *Escherichia coli*. ΔE160, S179F and K273E are produced by missense mutations in the *TNNT2* gene [28], [29], [30]. TnT Δ14 and Δ28 + 7 are the two C-terminal truncation products produced by the splice-site mutation _int_15 G1 > A [31]. All of these mutations increased Ca^2+^-sensitivity relative to donor heart troponin as previously found with *ACTC* E99K and *TPM1* E180G [7], [8], [9], [10], [11], [13], [14], [17], [32] (Fig. 4A). All of these mutations fully uncoupled the relationship between Ca^2+^-sensitivity and TnI phosphorylation, Fig. 3, Fig. 4B, Table 3.

### 3.4 EGCG can recouple the uncoupled HCM related mutations in TnT

**Fig. 5:**
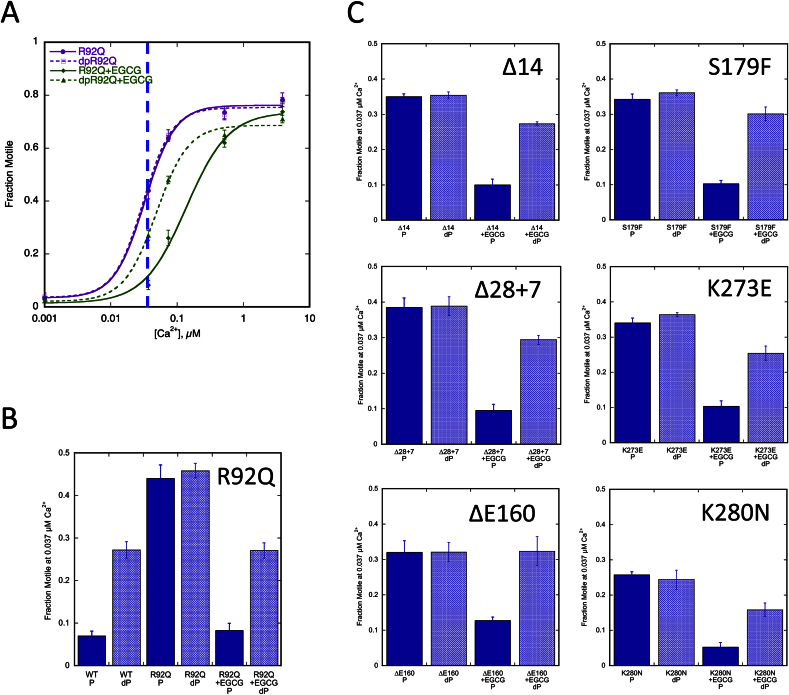
**Effect of EGCG on the different HCM-causing TnT mutations**. A. Effect of EGCG on Ca^2+^ regulation of thin filaments containing the TnT R92Q HCM-causing mutation. Fraction of filaments motile, measured by IVMA, is plotted against [Ca^2+^] for representative experiments. Solid lines and points, phosphorylated troponin; dotted lines and open points, unphosphorylated troponin. Error bars represent SEM of four measurements of motility in the same motility chamber. Purple, TnT R92Q containing thin filaments; green, the presence of 100 μM EGCG. B. The effect of EGCG at a single Ca^2+^ concentration (0.037 μM, shown by the blue dotted line in Fig. 5A) is plotted for TnT R92Q. C. The effect at 0.037 μM Ca^2+^ is plotted for the six other HCM-causing TnT mutations.

In a previous study we found that the uncoupling effect of DCM-causing mutations could be reversed by Epigallocatechin-3-gallate (EGCG) and related compounds *in vitro* and in myofibrils [17]. EGCG could also restore coupling to the HCM mutations, *TPM1* E180G and *ACTC* E99K. We therefore investigated whether EGCG could restore coupling to HCM related mutations in TnT. Fig. 5A shows that modulation of Ca^2+^-sensitivity by TnI phosphorylation was fully restored to thin filaments containing the TnT R92Q mutation. This is shown by a shift of both curves to the right on addition of 100 μM EGCG with the phosphorylated curve shifting more to bring back the difference in Ca^2+^-sensitivity due to phosphorylation (as shown in Fig. 1C). This can also be shown by plotting the difference in fraction motile at a fixed Ca^2+^ concentration (Fig. 5B). The other mutations were tested at a fixed concentration of 0.037 μM Ca^2+^ and likewise showed restoration of modulation of Ca^2+^-sensitivity by TnI phosphorylation (Fig. 5C). Thus EGCG is capable of restoring coupling to every mutant troponin that is uncoupled both in DCM and HCM.

## 4 Discussion

### 4.1 Regulatory properties of the homozygous TnT K280N mutant patient tissue sample

This sample enabled us to study troponin with an HCM related mutation in TnT in the context of the patient's heart. Contractility in the same sample has also been studied in skinned muscle fibres and in single myofibrils [33]. Both the isolated troponin and the skinned muscle fibres indicated that myofilament Ca^2+^-sensitivity was not very different from donor heart controls: the two were indistinguishable in IVMA whilst TnT K280N patient skinned muscle had a 20% higher Ca^2+^-sensitivity in skinned fibres. The studies in myofibrils indicated that the kinetics of contraction at saturating Ca^2+^ were altered by the mutation, with k_ACT_ and slow k_REL_ both being increased compared to donor heart myofibrils, similar to a previous study with an HCM mutation in myosin, however, Ca^2+^ dependence was not measured [34].

Exchange experiments permit the TnT K280N mutation to also be studied in troponin with a recombinant mutation introduced into ‘normal’ donor heart troponin. The regulatory properties are different, indicating that secondary changes have occurred to the troponin in the HCM patient's heart as previously found by Bayliss et al. [23]. Recombinant human cardiac TnT K280N increased myofilament Ca^2+^-sensitivity 1.7-fold in the IVMA, as is common with HCM-causing mutations (Fig. 2, Table 2). It is interesting that the effect of 95% mutation, a similar level to that found in the patient, and 45% mutation produced a similar Ca^2+^-sensitivity shift, suggesting that this mutation was dominant negative in common with most HCM-causing mutations. However, there is no data available on individuals with a heterozygous TnT K280N mutation that would allow us to confirm this conclusion.

In contrast to measurements of Ca^2+^-sensitivity, the effect of the mutation on the kinetics of myofibrillar force production and relaxation at saturating Ca^2+^ concentrations showed that exchange of recombinant human cardiac TnT K280N into donor and also of wild-type troponin into TnT K280N patient myofibrils gave fully reversible effects [33]. Since IVMA measures unloaded movement at 29 °C, whilst studies on myofibrils or permeabilised cells measure isometric force only at saturating Ca^2+^ at 15 °C, the studies are not directly comparable as we discussed previously [17] and therefore not contradictory.

The patient sample is unusual since the HCM phenotype was found with a high level of TnI phosphorylation, equivalent to donor heart muscle. Previously HCM was studied in tissue from septal myectomies that always had a very low level of phosphorylation of both TnI and MyBP-C [24], [35], [36]. Since the patient had a myectomy prior to the transplant it seems likely that the operation was beneficial to the septum, as determined by the TnI phosphorylation level, but the disease-causing mutation remained and it was still uncoupled (as is the case with DCM). The myectomy did not prevent subsequent heart failure, perhaps due to diastolic dysfunction.

When we tested the Ca^2+^ sensitivity at high and low levels of phosphorylation there was no difference, i.e. the troponin was uncoupled. This is similar to findings in myectomy samples where uncoupling was independent of the HCM-causing mutations [23]. When we repeated the test with donor troponin exchanged with recombinant human cardiac TnT K280N, Ca^2+^-sensitivity was still uncoupled, so in this case a single point mutation in TnT is sufficient to uncouple Ca^2+^-sensitivity from TnI phosphorylation.

### 4.2 Uncoupling is a common feature of HCM related mutations in TnT

An important question addressed in this manuscript is whether all mutations in TnT that are associated with HCM are uncoupled. We therefore examined six additional well-characterised HCM-linked mutations in TnT and found that they all increased Ca^2+^-sensitivity as previously reported [22], [37], [38], [39], [40], and all exhibited uncoupling of Ca^2+^-sensitivity from TnI phosphorylation levels. The mutant TnT R92Q troponin extracted from transgenic mice, expressed at 65% [19], and the five TnT mutations studied using recombinant troponin were all fully uncoupled. Therefore, mutations in TnT join mutations in TnI (R145G and R21C, measured by ATPase, IVMA and in myofibrils and skinned fibres and K206Q, measured by ATPase and IVMA), TnC (L29Q, measured by IVMA and ATPase and Y5H, I148V and M103I, measured in skinned papillary muscle), actin (E99K, measured by IVMA and in myofibrils) and tropomyosin (E180G, measured by IVMA) as mutations demonstrated to cause uncoupling *in vitro* [7], [8], [9], [10], [11], [13], [14], [17], [32], [41]. Thus, for HCM-causing mutations in genes coding for sarcomeric thin filament proteins, Ca^2+^-sensitisation together with uncoupling *in vitro* is likely to be a common consequence and they may contribute to the HCM phenotype.

**Fig. 6:**
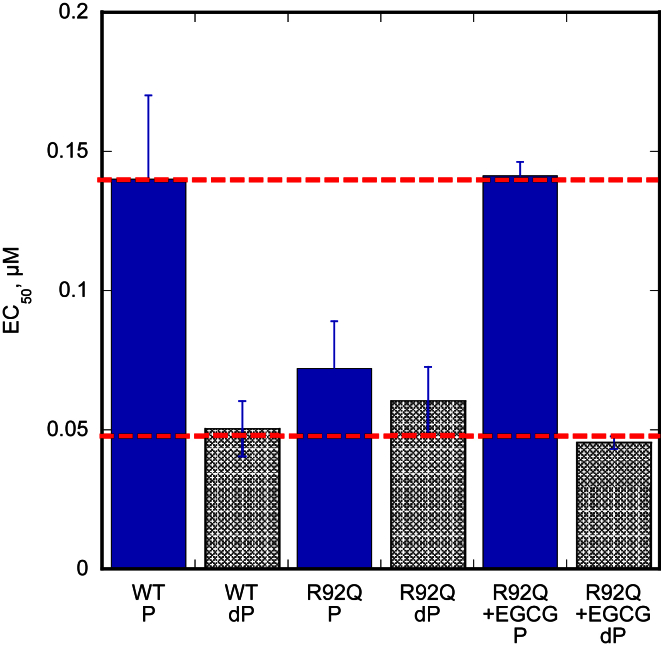
**Effect of EGCG on the HCM-causing mutation TnT R92Q**. Summary of the HCM-causing mutation TnT R92Q, EC_50_ values from replicate paired experiments are plotted. Error bars represent SEM of four measurements of motility in the same motility chamber. The TnT R92Q mutation causes the thin filaments to be uncoupled and EGCG re-couples the system and brings back the difference in Ca^2+^-sensitivity seen with wild-type (or donor) thin filaments.

We recently demonstrated that EGCG and related compounds could restore coupling to DCM-causing mutations in thin filament proteins [17]. We now show that EGCG can also restore coupling to all the uncoupled HCM-causing TnT mutations. Re-coupling by EGCG was also observed for *ACTC* E99K and *TPM1* E180G suggesting a common mechanism [17]. In fact the combination of Ca^2+^-desensitisation and re-coupling reverses both the abnormalities found in troponin with an HCM mutation. This is illustrated for the TnT R92Q mutation where the EGCG treatment fully restores motility to the levels of wild-type Ca^2+^-sensitivity and phosphorylation dependence (Fig. 6). This suggests a potential use for re-coupling compounds based on EGCG as treatment for HCM.

## Funding

This work was supported by grants from the British Heart Foundation (RG/11/20/29266 and FS/12/24/29568) and the Seventh Framework Program of the European Union ‘BIG-HEART’ (grant agreement 241577).

## Conflict of interest statement

The authors declare no conflict of interest.
