# Supplementary material for: Mutations in troponin T associated with Hypertrophic Cardiomyopathy increase Ca2+-sensitivity and suppress the modulation of Ca2+-sensitivity by troponin I phosphorylation
Source: Arch Biochem Biophys. 2016 Jul 1;601:113–20. doi: 10.1016/j.abb.2016.03.027 (PMC4909753; doi:10.1016/j.abb.2016.03.027)
Supplement: Supplementary file 1 [file mmc1.pptx]

## Slide 1
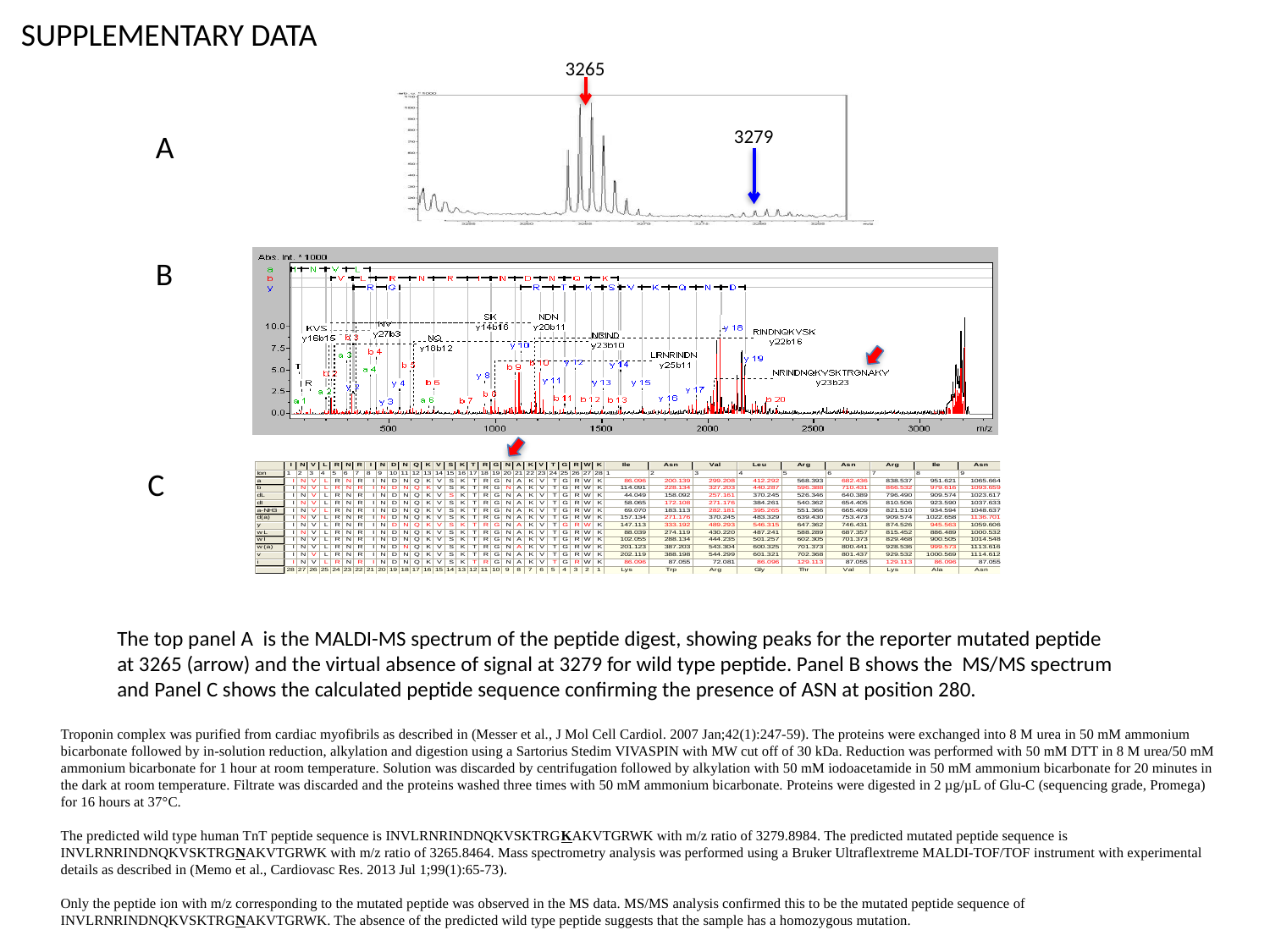

SUPPLEMENTARY DATA
3265
3279
A
B
C
The top panel A is the MALDI-MS spectrum of the peptide digest, showing peaks for the reporter mutated peptide at 3265 (arrow) and the virtual absence of signal at 3279 for wild type peptide. Panel B shows the MS/MS spectrum and Panel C shows the calculated peptide sequence confirming the presence of ASN at position 280.
Troponin complex was purified from cardiac myofibrils as described in (Messer et al., J Mol Cell Cardiol. 2007 Jan;42(1):247-59). The proteins were exchanged into 8 M urea in 50 mM ammonium bicarbonate followed by in-solution reduction, alkylation and digestion using a Sartorius Stedim VIVASPIN with MW cut off of 30 kDa. Reduction was performed with 50 mM DTT in 8 M urea/50 mM ammonium bicarbonate for 1 hour at room temperature. Solution was discarded by centrifugation followed by alkylation with 50 mM iodoacetamide in 50 mM ammonium bicarbonate for 20 minutes in the dark at room temperature. Filtrate was discarded and the proteins washed three times with 50 mM ammonium bicarbonate. Proteins were digested in 2 µg/µL of Glu-C (sequencing grade, Promega) for 16 hours at 37°C.
The predicted wild type human TnT peptide sequence is INVLRNRINDNQKVSKTRGKAKVTGRWK with m/z ratio of 3279.8984. The predicted mutated peptide sequence is INVLRNRINDNQKVSKTRGNAKVTGRWK with m/z ratio of 3265.8464. Mass spectrometry analysis was performed using a Bruker Ultraflextreme MALDI-TOF/TOF instrument with experimental details as described in (Memo et al., Cardiovasc Res. 2013 Jul 1;99(1):65-73).
Only the peptide ion with m/z corresponding to the mutated peptide was observed in the MS data. MS/MS analysis confirmed this to be the mutated peptide sequence of INVLRNRINDNQKVSKTRGNAKVTGRWK. The absence of the predicted wild type peptide suggests that the sample has a homozygous mutation.
